# Linking for Impact: Enhancing Linked Data Infrastructure to Meet Clinical Trials Sector Needs

**DOI:** 10.23889/ijpds.v9i5.2597

**Published:** 2024-09-18

**Authors:** Kate Miller, Felicity Flack

**Affiliations:** 1 Population Health Research Network

## Background

Australia possesses valuable population health data, yet its potential for advancing medical product development remains largely untapped. Transformative growth in the use of linked data in clinical trials will support Australia’s therapeutic development sector to be more responsive in the development of new therapeutics and the monitoring and surveillance of their safety and effectiveness.

## Methods

We conducted interviews with clinical trialists and data linkage experts to understand the current state and awareness of linked data within the clinical trials sector, identify existing pain points in the linkage process, and explore opportunities for improving visibility and access for the clinical trials sector.  Researchers represented all stages of the medical product development pipeline and had a diverse range of experience with linked data.

## Results

Interviews revealed numerous barriers hindering the clinical trials sector from effectively using linked data, limiting its impact. Barriers included a lack of awareness, the complexity of the application and approval process, and lengthy delays in accessing data.

Participants readily recognised several ways that linkage of real-world data could be applied to their work in clinical trials, with pre-recruitment data, measurements of primary and secondary endpoints, and health economic analysis being the most consistently noted use cases.

## Conclusion

To optimise the use of linked data to support clinical trials and medical product development in Australia, increased awareness among the therapeutic development sector regarding available real-world data and its potential is needed urgently. Lowering the barriers to accessing linked data will enhance health services and patient outcomes across Australia.

